# Functional Characterization of a Small-Molecule Inhibitor of the DKK1-LRP6 Interaction

**DOI:** 10.5402/2012/823875

**Published:** 2012-01-23

**Authors:** Sara Iozzi, Rosaria Remelli, Barbara Lelli, Daniela Diamanti, Silvia Pileri, Luisa Bracci, Renza Roncarati, Andrea Caricasole, Simonetta Bernocco

**Affiliations:** ^1^Pharmacology Department, Sienabiotech S.p.A, Strada del Petriccio e Belriguardo 35, 53100 Siena, Italy; ^2^Dipartimento di Biotecnologie, Università Degli Studi di Siena, Via Fiorentina 1, 53100 Siena, Italy; ^3^High-throughput Screening Unit, Center for Genomic Regulation, Dr. Aiguader, 88, 08003 Barcelona, Spain

## Abstract

*Background*. DKK1 antagonizes canonical Wnt signalling through high-affinity binding to LRP5/6, an essential component of the Wnt receptor complex responsible for mediating downstream canonical Wnt signalling. DKK1 overexpression is known for its pathological implications in osteoporosis, cancer, and neurodegeneration, suggesting the interaction with LRP5/6 as a potential therapeutic target. *Results*. We show that the small-molecule NCI8642 can efficiently displace DKK1 from LRP6 and block DKK1 inhibitory activity on canonical Wnt signalling, as shown in binding and cellular assays, respectively. We further characterize NCI8642 binding activity on LRP6 by Surface Plasmon Resonance (SPR) technology. *Conclusions*. This study demonstrates that the DKK1-LRP6 interaction can be the target of small molecules and unlocks the possibility of new therapeutic tools for diseases associated with DKK1 dysregulation.

## 1. Background

DKK1 is a 29 KDa secreted protein belonging to the Dickkopf (DKK) family [1], which comprises four main glycoproteins in vertebrates (DKK1-4) [2]. DKK1 has been identified as a potent inhibitor of the canonical Wnt signaling due to its ability to bind to the Wnt coreceptor LRP5/6, thus blocking the canonical Wnt/*β*-catenin pathway [3].

Canonical Wnt pathway activation is initiated by the direct binding of the Wnt glycoprotein to Frizzled (Fz) membrane receptor and to the LRP5/6 coreceptor [4–6]. In the absence of Wnt, *β*-catenin undergoes phosphorylation-dependent ubiquitination and degradation [7, 8]. Wnt-mediated assembly of the activated Fz-LRP5/6 receptor complex is followed by the recruitment of the axin-GSK3*β* to the plasma membrane, resulting in the reduction of the phosphorylation and degradation of *β*-catenin [9, 10]. Stabilized *β*-catenin accumulates in the cytoplasm and translocates to the nucleus, where it interacts with DNA-bound TCF-LEF proteins and activates the transcription of target genes [11].

The Wnt pathway is involved in many stages of invertebrate and vertebrate development and in adult tissue homeostasis [8, 12]. Dysfunction within the Wnt/*β*-catenin signaling cascade has been associated with many human pathologies [8, 13], such as cancer [14–17] and bone disease [18, 19]. LRP5-activating mutations are mainly associated with high-bone mass, while loss-of-function mutations on LRP5 are linked to bone degeneration and osteoporosis [20, 21]. The inhibition of Wnt signaling by DKK1 has been related to bone degeneration processes and reduced bone mass [22].

In the central nervous system, DKK1 has been associated with the pathophysiology of neuronal degeneration in Alzheimer disease (AD) [23–26]. DKK1 expression is increased in cortical neurons exposed to *β*-amyloid peptide (*β*-AP), where its aberrant expression is responsible for hyperphosphorylation of tau in neurons challenged with *β*-AP. Moreover, increased DKK1 expression was also observed in degenerating neurons in brain samples of AD patients [27]. DKK1 was shown to be neurotoxic when locally infused into brain regions where neurodegenerative processes associated with brain ischemia or AD normally take place [28]. Moreover, DKK1 is also associated with neuronal death in cellular and animal models of excitotoxic/ischemic neuronal death [29], and the treatment of ischemic animals with DKK1 antisense oligonucleotides protects hippocampal neurons against ischemic damage and cultured cortical neurons against NMDA toxicity. For these reasons, the DKK1-LRP6 interaction can be considered as a potentially interesting therapeutic intervention point and DKK1 a potential drug target for the treatment of bone and neurodegenerative disorders.

A small molecule (NCI8642) has been described as an inhibitor of the interaction between DKK1 and one of its receptors (LRP5), as well as an inhibitor of DKK1 activity in reducing Wnt/*β*-catenin signaling activation [30]. In this study, we sought to further characterise NCI8642 activity, using biochemical and biophysical approaches, and to extend its characterization in relation to LRP6, given the prominent expression in brain of LRP6 and its relevance to the neurodegenerative diseases field.

## 2. Methods

### 2.1. Cell Culture, Chemicals, Antibodies, and Plasmids

Human Embryonic Kidney cells (HEK293) were obtained from the German Collection of Microorganisms and Cell Cultures. Wnt3a L-cells and parental L-cells were obtained from ATCC. All cell lines were cultured in DMEM supplemented with FBS and Glutamax (Gibco). NCI8642 (gallocyanine) was purchased from Sigma-Aldrich. Passive Lysis Buffer and the AttoPhos substrate were purchased from Promega. The monoclonal antibody against *β*-catenin was obtained from BD Biosciences, the polyclonal goat anti-DKK1 antibody from R&D, while the fluorescently conjugated secondary antibodies were all from Invitrogen. Commercial purified recombinant human DKK1 and LRP6-Fc were purchased from R&D.

### 2.2. Plasmids and Recombinant Cell Lines

The DNA encoding the full-length human LRP6 sequence was cloned into the pCDNA3.1/Zeo(+) expression vector (Invitrogen). The DNA encoding the human DKK1 sequence was cloned into pCDNA6.2/cLumio-DEST (Invitrogen). The sequence encoding the secreted alkaline phosphatase from Clontech was amplified by PCR and inserted at the C-terminus of DKK1 sequence into the pCDNA6.2/cLumio-DEST-DKK1 plasmid. The luciferase reporter plasmid p4TCF-Luc comprises four copies of a TCF-responsive element upstream of a TATA element luciferase coding sequence transcriptional unit [31]. All cell lines were cultured in DMEM supplied with 10% FBS, 2 mM Glutamax, 100 units/mL penicillin, 100 *μ*g/mL streptomycin, and the appropriate selection antibiotic (50 and 100 *μ*g/mL Zeocine for PC12-TcfLuc and HEK293-LRP6, resp., 10 *μ*g/mL blasticidine for HEK293-DKK1, 0.4 mg/mL G418 for Wnt3a L-cells, 20 *μ*g/mL HEK293-AP-DKK1). For the generation of the stable cell lines, HEK293 or PC12 cells were transfected with Fugene 6 (Roche) and subjected to antibiotic selection 24 hours after transfection. Clone picking was done by ring cloning or dilution cloning. Clones were selected using immunofluorescence (HEK293-DKK1, HEK293-LRP6), AP enzymatic assay on the culture medium (HEK293-AP-DKK1), or measurement of luciferase activity upon stimulation with Wnt3a CM (PC12-TCF-Luc).

### 2.3. Production of Conditioned Media

For the production of DKK1 and AP-DKK1 conditioned media (CM), HEK293 cells stably expressing the appropriate construct were seeded into a 150-cm^2^ flasks at half confluence. The day after, the culture medium was replaced with Optimem. Media were collected 96 h after seeding, sterile filtered, and stored at −20°C in aliquots. The control conditioned Optimem was obtained in the same way using the parental HEK293 cells. Wnt3a CM and its control medium were produced as described [32]. For the functional studies (*β*-catenin translocation and TCF-Luc reporter assay), Wnt3a CM was used at a 1 : 2 dilution. Each batch of DKK1 CM was functionally titrated in order to determine the volume of CM that gives about 50% inhibition of Wnt signaling. From dot blot analysis (data not shown), the concentration of DKK1 in the DKK1 CM was estimated to be about 1 *μ*g/mL. For the quantitative AP-DKK1 binding assay, AP-DKK1 CM was diluted to obtain a phosphatase activity of 20 mU/mL.

### 2.4. AP-DKK1 Binding Assay

For the immunofluorescence detection of cell-bound DKK1, LRP6-293 cells were seeded onto poly-D-lysine-coated coverslips 24 hours before the experiment and incubated with DKK1 or AP-DKK1 CM diluted in cell culture medium for 2 hours at 4°C. When indicated, 100 *μ*M NCI8642 (or 1% DMSO) was added alongside DKK1 CM. After washing, the cells were fixed in 3% paraformaldehyde (15 min, room temperature), blocked with PBS/0.1% BSA, and incubated with the primary goat-anti-DKK1 antibody, and fluorescent secondary antibody diluted into blocking solution. Cell nuclei were counterstained with Hoechst 33342 dye. Images were collected using a Zeiss LSM 510 Meta confocal microscope. For the quantitative AP-DKK1 binding assay, the protocol was adapted from Zhang et al. [33]. The AP activity in the cell lysate was measured with Attophos AP fluorescent substrate, according to the manufacturer's instructions.

### 2.5. SPR Assay

SPR was performed using a Biacore T100 (GE Healthcare). Where not specified, experiments were conducted at 25°C. Bia T100 Evaluation Software Version 2.0.3 was used to analyze data. Kinetic constants were calculated by nonlinear fitting to the association and dissociation curves according to the manufacturer's instructions. Apparent equilibrium dissociation constants (K_D_) were then calculated as the ratio of k_d_/k_a_.

All immobilization steps were performed at a flow rate of 5 *μ*L/min using HBS-EP+ buffer (10 mM HEPES buffer, pH 7.4, containing 0.15 M NaCl, 3 mM EDTA, and 0.05% (v/v) P20 surfactant). Full-length human LRP6-Fc tagged (R&D Systems) was immobilized via amine coupling on a flow cell of a CM3 sensor chip. 3000 Resonance Units (RUs) were measured at the end of the immobilization procedure.

For the indirect capture of LRP6, Protein A was covalently coupled via amine immobilization on CM3 chip. LRP6-Fc capture on Protein A coated surface was performed by injecting the protein for 180 sec at the concentration of 0.5 *μ*g/mL in HBS-EP+ with a flow rate of 10 *μ*L/min.

Control sensorgrams, obtained on an empty flow cell where the coupling reaction had been conducted in the presence of coupling buffer alone, were always subtracted from binding responses. This allowed subtraction of nonspecific binding, which might be generated by residual charged negative groups, which had not been neutralized by the coupling procedure. Bovine Serum Albumine (BSA) was immobilized via standard amine coupling procedures on a flow cell of a CM3 sensor chip to obtain the nonrelated control flow cell.

For binding and kinetic assays, DKK1 (R&D Systems), diluted in HBS-EP+ at concentration ranging from 3 to 54 nM, was injected for 180 sec at the flow rate of 20 *μ*L/min over LRP6 either directly immobilized or captured via Protein A as described before. For NCI8642 binding and kinetic assays, the compound was diluted in HBS-EP+ buffer at concentration ranging from 3 to 50 *μ*M and injected over LRP6 surface for 60 sec at the flow rate of 30 *μ*L/min. HBS-EP+ Buffer pH 7.4 0.2% DMSO was used as running buffer. 0.2% DMSO was added to HBS-EP+ when performing the experiments with NCI8642 compound, and DMSO correction module of the software was used to compensate for refractive index change induced by DMSO.

For inhibition analysis, DKK1 (54 nM, diluted in HBS-EP+ Buffer pH 7.4) was incubated with NCI8642 at concentration from 5 to 20 *μ*M. After 1 hour at 25°C, DKK1 was injected over immobilized LRP6 for 240 s at the flow rate of 10 *μ*L/min.

### 2.6. TCF-Luciferase Reporter Assay

PC12-TCF-Luc cells were seeded at the density of 100,000 cells/well into black-walled clear-bottom 96-well plates. After 24 hours, culture medium was replaced with 50 *μ*L DKK1 CM diluted as appropriate in Optimem. Either DMSO or NCI8642 was added at the indicated final concentration. After one-hour incubation (37°C), the cells were stimulated by adding 50 *μ*L of Wnt3a CM (50% of the final volume) and supplemented with either DMSO or NCI8642 at the indicated final concentration (final DMSO concentration 1%). After 24 hours of incubation, the luciferase activity was measured with Steady Lite substrate (Perkin-Elmer), according to the manufacturer's instructions. For each condition 3, replicates were averaged.

### 2.7. *β*-Catenin Translocation Assay

L-cells were seeded into black-walled clear-bottom 96-well plates at a density of 10,000 cells/well. After 24 hours, culture medium was replaced with 50 *μ*L DKK1 CM diluted as appropriate in Optimem. Either DMSO or NCI8642 was added at the indicated final concentration. After one-hour incubation (37°C), the cells were stimulated by adding 50 *μ*L Wnt3a CM supplemented with either DMSO or NCI8642 at the indicated final concentration (final DMSO concentration 1%). After two-hour stimulation (37°C), the cells were fixed in 3% paraformaldehyde (10 min, room temperature), permeabilized (0.2% Triton X-100 in PBS, 10 min, room temperature), and blocked with 0.1% BSA in PBS. The cells were then incubated for 2 hours at room temperature with the primary anti-*β*-catenin antibody (1 : 500), followed by the secondary AlexaFluor 555 goat-anti-mouse antibody (1 : 1000). Cell nuclei were counterstained with Hoechst 33342 dye. Images were captured with the BD Pathway 435 Imaging system (20x magnification). Each condition was tested in triplicates (nine fields per well). Images were analysed using BD Attovision software. The ratio of the nuclear and cytoplasmic intensity of *β*-catenin staining was calculated for each cell and averaged within the well.

## 3. Results

### 3.1. NCI8642 Inhibits DKK1 Binding to LRP6 at the Cell Surface

**Figure 1:**
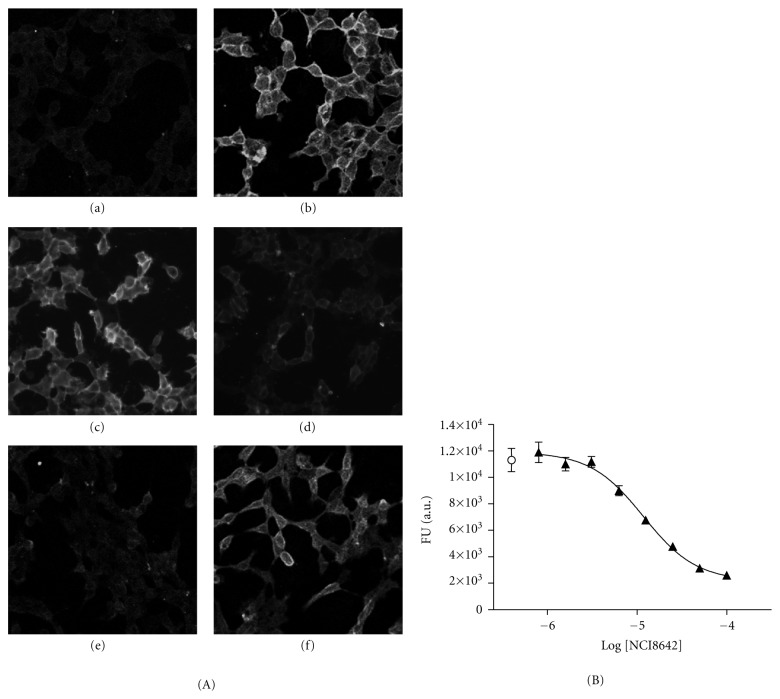
NCI8642 is able to abolish DKK1-LRP6 interaction at the cell surface. (A) Immunostaining of DKK1 on HEK293 cells (panel a), on HEK293-LRP6 cells (panel b), on HEK293-LRP6 cells in the presence of 1% DMSO (panel c), and on HEK293-LRP6 cells in the presence of 100 *μ*M NCI8642 (panel d). Immunostaining of AP-DKK1 on HEK293-LRP6 cells (panel e and f). The cell-bound DKK1 was detected with anti-DKK1 antibody. (B) Enzymatic quantification of AP-DKK1 binding to HEK293-LRP6 cells, in the presence of increasing concentrations of NCI8642.

NCI8642 was originally characterized as an inhibitor of the LRP5/DKK1 interaction [30]. In order to determine if NCI8642 was also able to disrupt the binding of DKK1 to LRP6, a recombinant HEK293 cell line stably expressing LRP6 was generated (HEK293-LRP6). An additional recombinant HEK293 cell line was established for the production of human recombinant DKK1 fused to the V5 tag at the C-terminus (HEK293-DKK1). When conditioned medium (CM) containing DKK1 was incubated on cells expressing LRP6 and the cell-bound DKK1 was detected by immunofluorescence, a strong membrane signal was observed in HEK293-LRP6 cells (Figure 1(A), panel b), while it was almost absent on the parental HEK293 cells (Figure 1(A), panel a). When DKK1 CM was supplemented with 100 *μ*M NCI8642, the binding of DKK1 (Figure 1(A), panel d) was reduced compared to the DMSO-treated control (Figure 1(A), panel c). In order to obtain a quantitative measure of the DKK1/LRP6 interaction, a construct coding for human DKK1 fused to a secreted domain of alkaline phosphatase was generated (AP-DKK1) and used to establish a stable HEK293 cell line secreting AP-DKK1 in the culture medium. The AP tag at the C-terminus does not impair DKK1 binding to LRP6, as shown by immunofluorescence of cell-bound AP-DKK1 on HEK293-LRP6 (Figure 1(A), panels e and f). The binding of AP-DKK1 to HEK293-LRP6, quantified by enzymatic AP assay, was reduced when AP-DKK1 was incubated in the presence of increasing concentrations of NCI8642. The binding of DKK1 was reduced in a concentration-dependent manner compared to DMSO sample (Figure 1(B)). The calculated IC_50_ was 14.6 *μ*M (pIC_50_ = 4.8, STDev 0.2, *n* = 10 independent experiments).

### 3.2. NCI8642 Binds to LRP6 and Inhibits DKK1-LRP6 Binding

**Figure 2:**
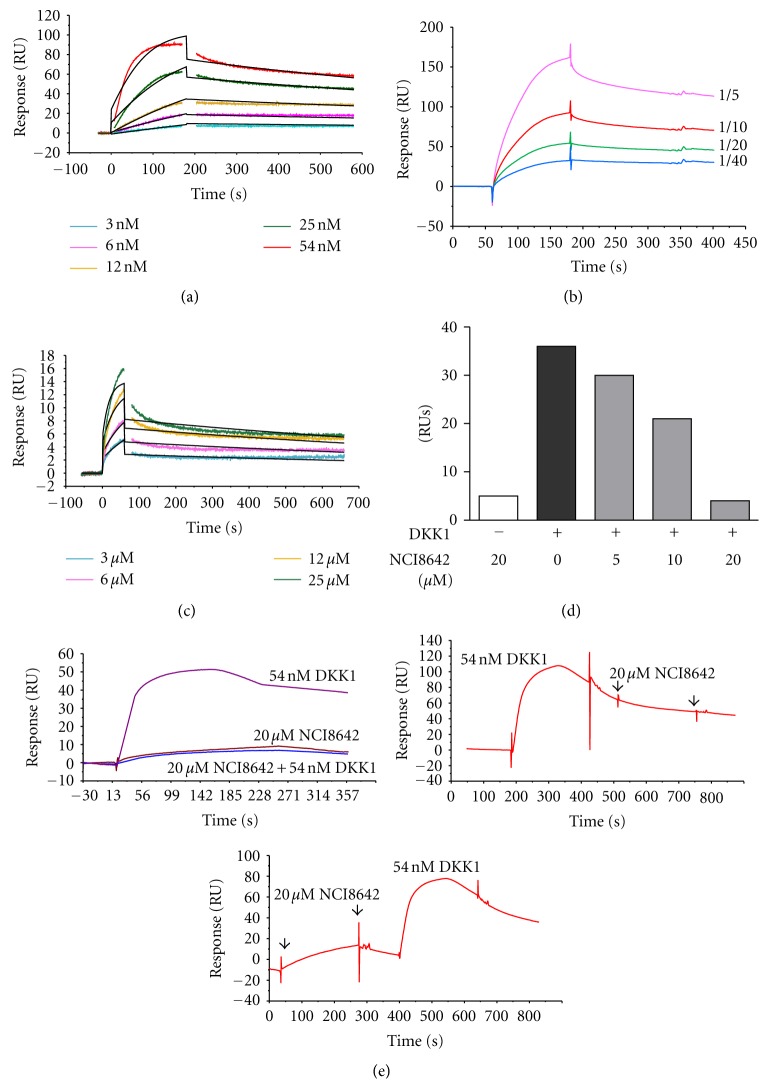
NCI8642 is able to abolish DKK1-LRP6 interaction *in vitro*. (a) Kinetic analysis of the interaction of DKK1 on LRP6. DKK1 was injected at the concentrations indicated in the legend (3–54 nM), for 180 sec at the flow rate of 30 *μ*L/min; regeneration was achieved with a very short pulse of 10 mM NaOH/1 M NaCl. A time of 500 seconds was allowed for spontaneous dissociation before regeneration step. Kinetic evaluation was achieved through the BIAevaluation software (k_a_ = 5.26*E* + 06  1/Ms, k_d_ = 1.83*E* − 03  1/s,   and  K_D_ = 3.49*E* − 10 M). (b) Binding of recombinant DKK1 CM injected at different dilutions over immobilized LRP6. (c) Kinetic analysis of NCI8642 on LRP6. NCI8642 was injected at the concentrations indicated in the legend (3–50 *μ*M), for 60 sec at the flow rate of 30 *μ*L/min. Kinetic evaluation of the small molecule was achieved through the BIAevaluation software (k_a_ = 2.86*E* + 03  1/Ms, k_d_ = 1.34*E* − 04  1/s,   and  K_D_ = 4.66*E* − 07 M). (d) Effect of NCI8642 on the interaction of DKK1 with LRP6. The compound was injected at a final concentrations of 5, 10, and 20 *μ*M; DKK1 concentration was 54 nM. The two molecules were injected together after 1-hour preincubation over the immobilized LRP6. The picture is representative of two single experiments. (e) Effect of NCI8642 on the interaction of DKK1 with LRP6. When DKK1 is injected together with 20 *μ*M NCI8642 after 1-hour incubation at 25°C, DKK1 binding on LRP6 is completely abolished (upper panel). Sequential injections of 20 *μ*M NCI8642 and 54 nM DKK1 do not inhibit the DKK1-LRP6 interaction (middle panel). 20 *μ*M NCI8642 is not able to displace DKK1 when injected at the end of DKK1 injection (lower panel).

The LRP6-DKK1 interaction was observed using surface plasmon resonance technology (SPR). When flowed over the surface of the sensor chip coated with recombinant LRP6-Fc, DKK1 bound to immobilized receptor both specifically and in a concentration-dependent manner (Figure 2(a), k_*on*_ 5.3 10^6^ M^−1^ s^−1^, k_*off*_ 1.8 10^−3^ s^−1^). Indirect capturing of LRP6-Fc on recombinant Protein A and direct LRP6-Fc covalent coupling via amine groups produced similar interaction with recombinant human DKK1 (K_D_ of 8.4 and 3.5 10^−10^ M, resp.), demonstrating that the covalent coupling of the receptor to the chip surface does not influence its binding site. The interaction constants obtained are in agreement with the literature data [1, 34, 35]. The LRP6-DKK1 interaction was also verified using recombinant DKK1 CM instead of commercial recombinant DKK1 (Figure 2(b)).

When we investigated whether NCI8642 was able to bind to the LRP6 receptor, we observed a consistent and effective binding signal that is not present in the control flow cell. The kinetic analysis of the interaction by injection of an NCI8642 dilution series (from 3 *μ*M to 50 *μ*M; Figure 2(c)) allowed the determination of a K_D_ of 4.7 10^−7^ M for NCI8642 on LRP6.

We also analyzed the effect of NCI8642 on the binding of DKK1 to LRP6, to determine whether the compound could interfere with the interaction between DKK1 and LRP6. We injected DKK1 and NCI8642 over LRP6 previously immobilized. When DKK1 was injected together with NCI8642 after 1-hour incubation at 25°C, we observed a consistent reduction of DKK1 binding on LRP6 with 5 *μ*M and 10 *μ*M compound, and even a complete binding inhibition at the concentration of 20 *μ*M (Figures 2(d) and 2(e)-upper panel). On the contrary, sequential injections of 20 *μ*M NCI8642 and 54 nM DKK1 did not inhibit the DKK1-LRP6 interaction. NCI8642 was not able to displace DKK1 when injected after it and DKK1 could still bind to LRP6 when injected after NCI8642 at the concentration of 20 *μ*M (Figure 2(e)-middle and lower panels).

### 3.3. NCI8642 Reverts DKK1-Mediated Inhibition of Wnt Signalling

**Figure 3:**
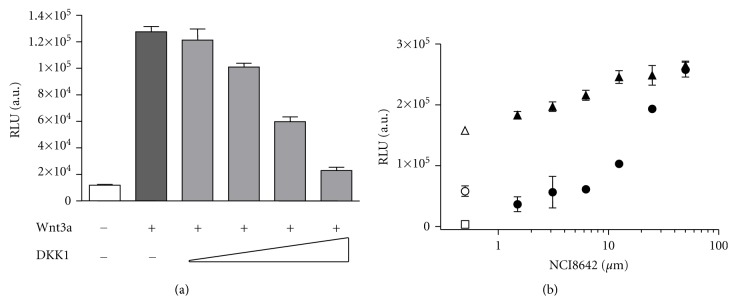
NCI8642 is able to revert DKK1 inhibition of the Wnt pathway. (a) Luciferase activity detected in PC12-TCF-Luc cells stimulated with Wnt3a alone and in presence of increasing volumes of DKK1 CM. (b) Effect of NCI8642 on Wnt3a and DKK1 treated cells. Open square: basal signal; open triangle: cells treated with Wnt3a CM; open circle: cells treated with Wnt3a, DKK1 CM, and DMSO 1%; close symbols: cells treated with serial dilutions of NCI8642 (up to 50 *μ*M), either in the presence (close circles) or in the absence (close triangles) of DKK1 CM.

To investigate the functional activity of NCI8642, we tested the compound in a TCF reporter-based assay in PC12 cells (neural crest-derived rat pheochromocytoma cells). TCF, similarly to LEF-1, is a downstream target transcription factor of the canonical Wnt-signaling pathway. A stable PC12 cell line expressing a TCF-dependent luciferase reporter was established (PC12-TCF-Luc). Wnt3a-mediated response was reverted by DKK1 CM in concentration-dependent manner (Figure 3(a)). For the purpose of compound testing, the dilution factor of DKK1 CM was chosen in order to have about 50% inhibition of Wnt signaling (Figure 3(b), open symbols). In the presence of exogenously added DKK1, NCI8642 was able to revert the DKK1-mediated inhibition. The effect was concentration-dependent and was reproducibly and significantly different from DMSO treatment at 25 *μ*M and 50 *μ*M, although higher concentrations of the compound were toxic to the cells and an IC_50_ value could not, therefore, be calculated.

**Figure 4:**
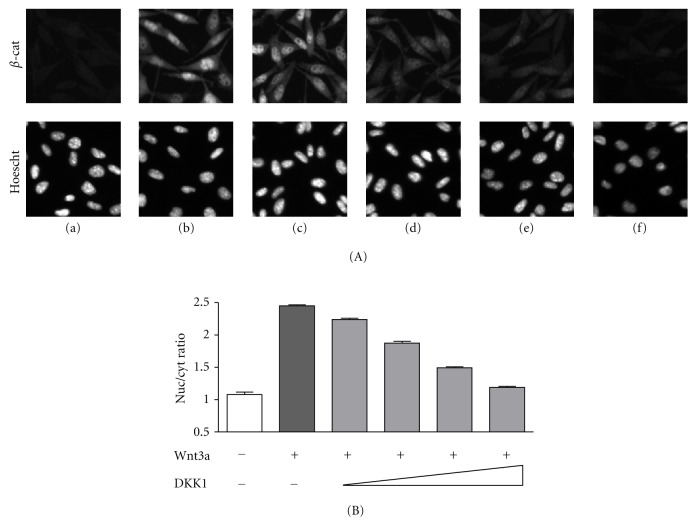
DKK1 inhibits Wnt3a-mediated *β*-catenin translocation. (A) Immunostaining of *β*-catenin in L-cells following stimulation with Wnt3a CM, either without DKK1 or in the presence of increasing volumes of DKK1 CM. Panel (a), unstimulated cells; panel (b), Wnt3a-stimulated cells; panels (c–f), Wnt3a-stimulated cells in presence of DKK1 CM dilutions. Cell nuclei counterstaining with Hoechst 33342 dye are shown for each panel. (B) Histogram representation of the nuclear/cytoplasmic intensity ratios corresponding to the cells shown in (A).

**Figure 5:**
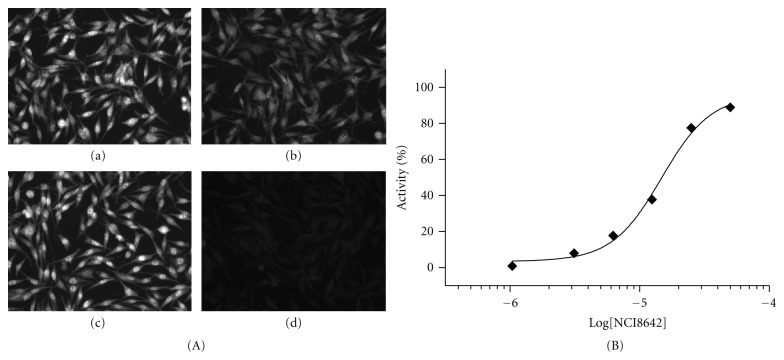
NCI8642 is able to compete with DKK1 in *β*-catenin translocation assay. (A) Panel a: Wnt3a-stimulated cells; panel b: cells treated with Wnt3a and DKK1; panel c: cells treated with Wnt3a, DKK1, and 50 *μ*M NCI8642; panel d: cells treated with 50 *μ*M NCI8642 only. (B) Concentration-response curve of NCI8642 (from 1 to 50 *μ*M) expressed as % of activity. The activity was defined as the ratio of nuclear/cytoplasmic intensity normalized between Wnt3a + DMSO treatment (100% activity) and Wnt3a + DKK1 + DMSO treatment (0% activity).

NCI8642 was also tested in an assay measuring the modulation of *β*-catenin intracellular accumulation and nuclear translocation. The assay was performed in L-cells, an established model system for studying Wnt signaling [36]. Upon treatment of L-cells with Wnt3a CM, the intracellular amount of *β*-catenin increases, with an enrichment in the nuclear compartment and a concomitant increase in the nuclear/cytoplasmic ratio of *β*-catenin intensity. In the sample treated with control CM, *β*-catenin is hardly detectable, with a nuclear/cytoplasmic ratio close to one. The Wnt3a-mediated effect is reverted by DKK1 CM in a concentration-dependent manner (Figures 4(A) and 4(B)). When NCI8642 was added to the cells together with DKK1, and then the cells were stimulated with Wnt3a, the effect of DKK1 was blocked. The measured IC_50_ of the compound is 12.6 *μ*M (pIC_50_ = 4.9 STDev 0.2, *n* = 5 independent measurements), in good agreement with the binding data and the reporter assay (Figures 5(A) and 5(B)).

## 4. Discussion

NCI8642 is a small molecule that has been recently identified as a specific inhibitor of LRP5-DKK1 interaction. Given the role of the Wnt signaling in many pathophysiological contexts, modulation of the Wnt pathway has become a valuable target in drug discovery [37]. The modulation of the pathway at the level of the cell surface poses the problem of targeting a protein-protein interaction, which might be challenging [38]. Recently, the LRP5-DKK1 interaction has been targeted with monoclonal anti-DKK1 antibodies [39, 40]. These antibodies were effective in increasing bone density *in vivo*, both in naïve normal growing female mice and in a model of postmenopausal osteoporosis. However, the lack of blood-brain penetration of the antibodies limits their application to peripheral indications such as osteoporosis. On the contrary, a small molecule blocking DKK1 inhibition would have the advantage of being potentially useful also for the treatment of CNS pathologies, such as neurodegeneration. Our study confirmed that NCI8642 acts as a DKK1 inhibitor, and we extended the finding to the interaction of DKK1 to LRP6. In fact, the small molecule caused the displacement of DKK1 from LRP6 overexpressed in HEK293 cells, as shown both by immunofluorescence and quantitative enzymatic assay. The displacement was also demonstrated in a cell-free context by using SPR technology, where NCI8642 blocks the binding of DKK1 to the immobilized LRP6. Interestingly, the compound was able to block DKK1 binding only when injected together with DKK1, and not when injected sequentially after DKK1, which is probably due to the high affinity of DKK1 for LRP6 compared with the micromolar affinity of NCI8642 for LPR6 (Figures 2(c) and 2(e)).

SPR technology also showed that NCI8642 interacts with the immobilized LRP6 with a reproducible and concentration-dependent binding in the absence of DKK1 (K_D_ 4.7 10^−7^ M). The interaction between NCI8642-LRP6 is highly specific, because no significant binding was observed on two control flow cells. When immobilized on the sensor chip, DKK1 lost the ability to bind LRP6, suggesting that the immobilization caused a modification of DKK1 structure-function, thus the direct interaction of NCI8642 to DKK1 was not measured.

NCI8642 is also functionally active in the inhibition of DKK1 activity on Wnt signaling mediated by LRP5/6 as demonstrated by the TCF-Luc and *β*-catenin assays in two different cellular backgrounds. The compound IC_50_ measured in the TCF-Luc and *β*-catenin assays, where LRP5/6 is expressed at endogenous, physiological levels, is comparable with the one obtained in the binding assay on the overexpressed LRP6. In the TCF-Luc assay, we observed a slight increase of the Wnt3a-mediated activation of the reporter gene, even in the absence of DKK1. The hypothesis that it could be due to the presence of endogenous DKK1 in these cells was not confirmed, since we could not detect it by western blot or by quantitative RT-PCR (data not shown). Nevertheless, this was not observed in the *β*-catenin translocation assay (data not shown), suggesting that it could be an artifact of the reporter assay, or a cell-line-specific effect. Although NCI8642 has been reported as a DKK1/LRP5 inhibitor, our data suggest that it is also effective on DKK1/LRP6 interaction. In fact, NCI8642 blocked DKK1 binding on LRP6, both in LRP6 overexpressing cells and in SPR analysis. This was also confirmed by functional studies, since only LRP6, but not LRP5, is expressed in PC12-TCF-Luc cell line [41], and LRP6 mRNA concentration is about 7 times that of LRP5 in L-cells, as assessed by qPCR (data not shown).

Our results suggest that it is possible to antagonize the DKK1-LRP6 interaction with a small molecule, and this leads to an enhancement of the Wnt signaling pathway. Although NCI8642 micromolar potency might be too low for its application in *in vivo* experiments, this compound can be considered as a reference small molecule for further drug development aiming at the inhibition of this therapeutically important protein-protein interaction.

## Abbreviations

### Abbreviations

LRP5/6: Low-density lipoprotein receptor-related protein 5 or 6
DKK1: Dickkopf 1
GSK3*β*: Glycogen synthase kinase beta
TCF/LEF: T-cell factor/lymphoid enhancer factor
SPR: Surface plasmon resonance
FU: Fluorescence units
RLU: Relative luminescence units
RU: Resonance units
AP-DKK1: Alkaline phosphatase-DKK1.
